# Difficult Management of a Rare Case of Distal Vaginal Atresia in an Adolescent

**DOI:** 10.7759/cureus.102218

**Published:** 2026-01-24

**Authors:** Karam Harou, Ilyas Benaissa, Abdoulhakim Abdou, Abderraouf Soumani

**Affiliations:** 1 Department of Obstetrics and Gynecology, Mohammed VI University Hospital Center, Cadi Ayyad University, Marrakesh, MAR; 2 Department of Obstetrics and Gynecology, Mohammed VI University Hospital Center, Marrakech, MAR

**Keywords:** hematocolpos, müllerian anomaly, primary amenorrhea, transverse vaginal septum, vaginal agenesis

## Abstract

A transverse vaginal septum is a rare congenital anomaly that can present with primary amenorrhea and cyclic pelvic pain due to obstructive Müllerian malformation. We report the case of a 16-year-old adolescent who presented with primary amenorrhea and eight months of progressive cyclic lower abdominal pain. Physical examination revealed normal secondary sexual characteristics. Hormonal evaluation confirmed normal hypothalamic-pituitary-ovarian axis function. Pelvic MRI demonstrated hematometra and hematocolpos with a thin, regular vaginal septum and an associated distal vaginal agenesis component.

Initial surgical correction was performed via vaginal approach, involving circumferential incision of the obstructive septum, controlled drainage of retained menstrual blood, complete excision of septal tissue, and end-to-end mucosal anastomosis. Despite initial resolution of symptoms and establishment of normal menstrual flow, the patient experienced recurrent obstruction at two months postoperatively, requiring vaginal dilation. At four months, she developed pelvic sepsis from infected hematocolpos, necessitating surgical revision with fibrotic tissue excision and attempted vaginal mold placement, which was poorly tolerated and removed after four days.

This case highlights the challenging nature of complete transverse vaginal septum management, particularly when associated with distal vaginal agenesis. Despite appropriate initial surgical correction, a significant risk of stenosis, recurrent obstruction, and infectious complications exists, necessitating close postoperative surveillance and readiness for multiple interventions to achieve satisfactory long-term outcomes.

## Introduction

Distal vaginal atresia is an uncommon congenital anomaly of the female genital tract resulting from abnormal formation of the distal portion of the vagina, which may be associated with TBX3/AXL pathways and renal or vertebral anomalies. Normal vaginal development occurs between the 11th and 20th weeks of gestation and requires coordinated interaction between the Müllerian ducts, Müllerian tubercle, and sinovaginal bulbs to achieve canalization of the vaginal lumen. Failure of this process leads to complete obstruction of the lower vaginal segment.

In contrast to complete vaginal agenesis, such as Mayer-Rokitansky-Küster-Hauser (MRKH) syndrome, individuals with distal vaginal atresia have a normally developed uterus and proximal vagina. The malformation is limited to the distal vagina, where a fibrous or membranous barrier prevents communication with the external genital tract despite otherwise normal internal reproductive anatomy.

This condition is exceedingly rare. Although Müllerian anomalies are reported in approximately 0.1-0.5% of females, distal vaginal atresia constitutes only a small fraction of these abnormalities, with an estimated occurrence of 1 in 10,000 to 15,000 individuals [1]. The scarcity of cases contributes to limited clinical experience and the absence of standardized management guidelines.

Symptoms typically become evident during adolescence at the expected onset of menstruation. Patients commonly present with primary amenorrhea accompanied by cyclic pelvic or abdominal pain, reflecting progressive retention of menstrual blood proximal to the obstruction and the development of hematocolpos or hematometra.

Diagnosis requires a high index of suspicion and is based on clinical evaluation supported by imaging. Pelvic ultrasound is often used as an initial investigation, while magnetic resonance imaging provides superior delineation of the level and extent of vaginal obstruction and associated pelvic findings.

Surgical correction remains the cornerstone of treatment and aims to restore vaginal patency while preserving future reproductive and sexual function. Delayed diagnosis may lead to complications such as endometriosis, infection, or fibrosis, highlighting the importance of early recognition and individualized management.

## Case presentation

A 16-year-old adolescent presented to our tertiary care center for primary amenorrhea associated with progressive cyclic pelvic pain. Her medical history was unremarkable, with normal growth and development throughout childhood. She reported monthly episodes of lower abdominal pain for the past eight months, of increasing intensity. She denied significant weight loss or gain, fever, or urinary disorders. Family history was negative for reproductive tract anomalies or genetic diseases. Secondary sexual characteristics were appropriate, with thelarche beginning at age 13.

Physical examination revealed a well-developed adolescent in good general condition. Abdominal palpation showed moderate suprapubic tenderness, without organomegaly. Gynecological examination revealed normal external genitalia, with breast development and pubic hair. A complete transverse vaginal septum was identified in the upper third of the vagina, with a significant retrovaginal collection suggestive of hematocolpos.

**Figure 1:**
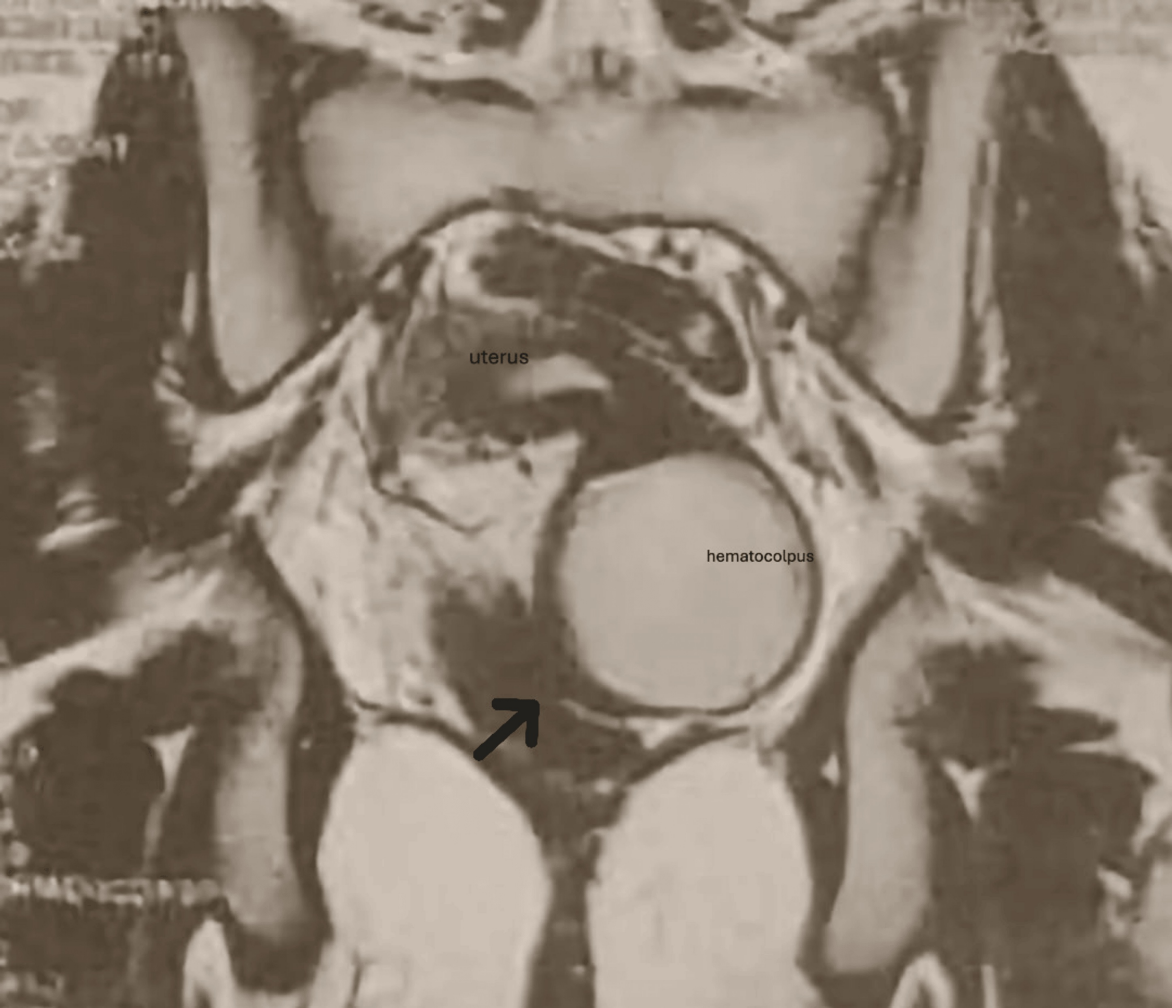
Pelvic MRI, T2-weighted coronal sequence, demonstrating hematometrocolpos with proximal vaginal distension The arrow indicates distal vaginal stenosis located downstream from the hematocolpos, consistent with distal vaginal atresia.

Hormonal evaluation showed normal gonadotropin and estradiol levels, confirming the integrity of the hypothalamic-pituitary-ovarian axis; therefore, the full hormonal profile was not detailed, as it did not contribute additional diagnostic information. Pelvic MRI revealed an anteverted uterus distended by hematometra, extending to the upper third of the vagina (Figure 1). A thin, regular vaginal septum was clearly visible, consistent with a complete transverse septum. Both ovaries appeared normal, and a small right paravaginal fluid collection measuring 13.4×8 mm was noted. Incidental findings included physiological inguinal and iliac lymph nodes.

After an in-depth multidisciplinary discussion and detailed counseling with the patient and her family (due to the country's ethical customs), surgical management was planned. Under general anesthesia, a vaginal approach was performed. Intraoperatively, an associated distal vaginal agenesis with an obstructive septum was carefully identified and circumferentially incised. Controlled drainage of the hematometra and hematocolpos collections was carried out, followed by complete excision of the septal tissue. Vaginal mucosa was reapproximated (end-to-end anastomosis) with absorbable sutures (Vicryl 3.0), with particular attention to hemostasis. The procedure was uneventful. 

The patient's initial recovery phase was unremarkable, with prompt resolution of her chronic pelvic pain complaints. Normal menstrual flow was established during the first postoperative month, suggesting adequate surgical correction of the anatomical defect.

**Figure 2:**
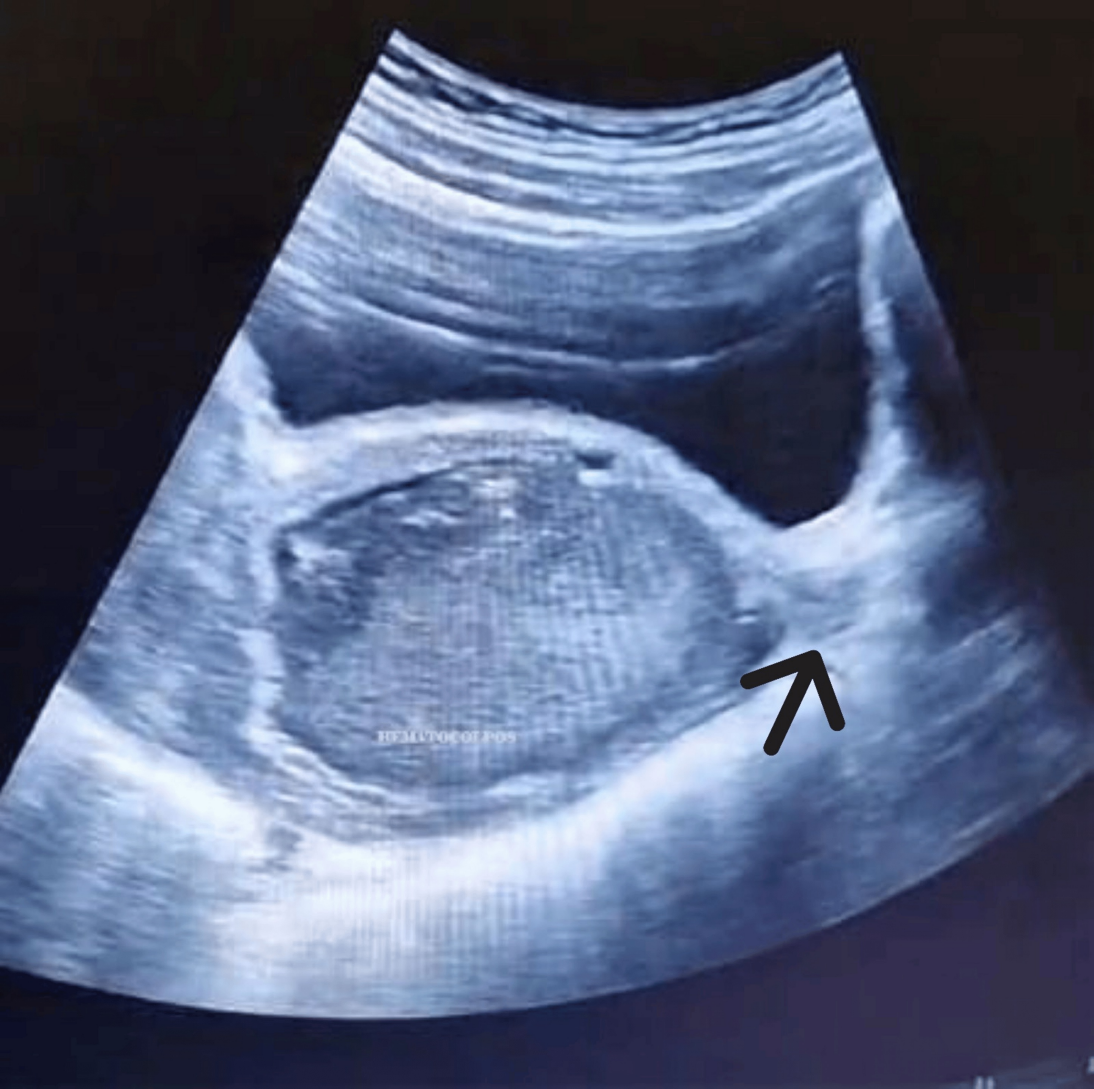
Ultrasonographic examination showing the arrow indicating the reformation of the obstructive lesion

However, the clinical course became complicated at two months when the patient developed recurrent abdominal pain. Pelvic ultrasonographic examination revealed reformation of the obstructive lesion (Figure 2), requiring therapeutic vaginal dilation as a corrective measure.

At four months postoperatively, the patient presented to our emergency department with fever and signs of pelvic sepsis. Clinical evaluation and imaging studies confirmed the presence of an infected hematocolpos secondary to menstrual blood retention. The treatment consisted of drainage combined with oral antibiotic therapy, with good clinical improvement. Three vaginal dilations were performed overall, including one during the drainage procedure.

**Figure 3:**
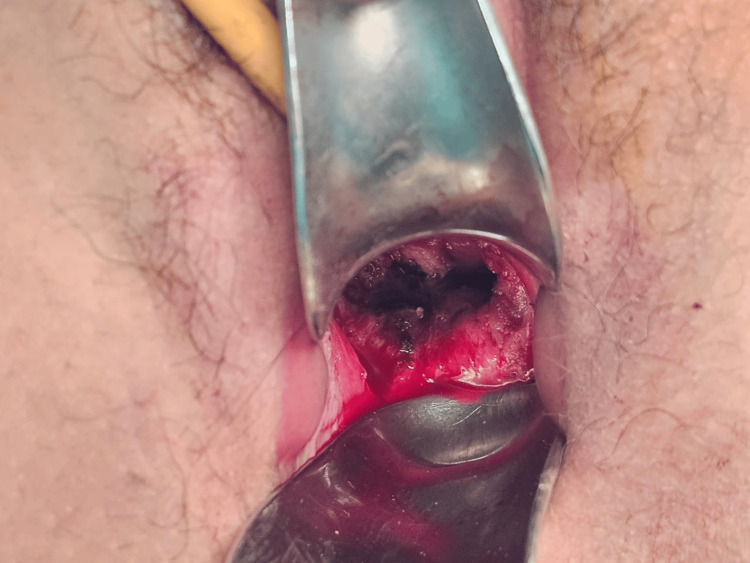
Vaginal scar fibrosis, recurrence of vaginal atresia

**Figure 4:**
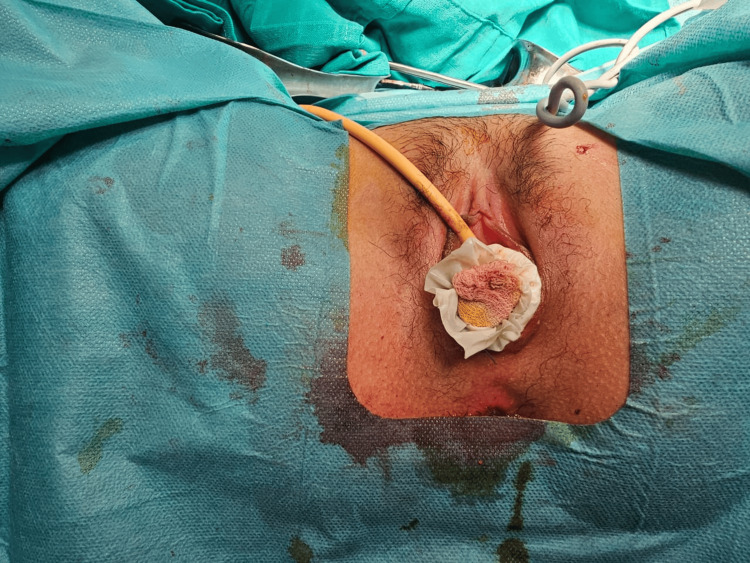
Insertion of a vaginal mold

Given the failure of conservative management and the severity of the infectious process, we opted for immediate surgical revision. The reoperation consisted of excision of the fibrotic tissue (Figure 3) and placement of a vaginal mold (Figure4). However, the patient did not tolerate the mold, and it was removed after four days due to discomfort and pain. No intraoperative complications were reported.

## Discussion

Hematometrocolpos secondary to congenital genital obstruction is a rare condition that represents a significant diagnostic and therapeutic challenge in adolescence. In our patient, distal vaginal atresia resulted in complete obstruction of menstrual outflow, leading to progressive accumulation of blood within the vagina and uterus and explaining the presentation with primary amenorrhea and cyclic pelvic pain. This case illustrates an uncommon etiology of hematometrocolpos and highlights the complexity of management when anatomical constraints are compounded by infection.

Only a limited number of cases of distal vaginal atresia complicated by hematometrocolpos have been reported in the literature [1]. Similar to previously published cases, our patient presented with typical clinical symptoms and imaging findings consistent with genital outflow obstruction. However, in contrast to some reports describing elective or extensive primary reconstructive procedures, the management in this case was strongly influenced by local infection and by limited tolerance to postoperative measures, which represented distinctive features compared with most published series.

Pathophysiology and embryology

From an embryological standpoint, distal vaginal atresia results from abnormal development or failed canalization of the distal vaginal sinus, preventing formation of a functional vaginal outlet [2,3]. In our case, this developmental anomaly translated clinically into complete obstruction, with secondary hematometrocolpos. Rather than reiterating the well-established pathophysiological mechanisms, the present discussion emphasizes how these mechanisms directly shaped the diagnostic and therapeutic strategy adopted for this patient.

Clinical and paraclinical diagnosis

In our patient, clinical examination was limited by pain, anatomical distortion, and signs of infection, making imaging essential for diagnosis and planning. Pelvic ultrasound revealed a fluid-filled vaginal and uterine mass suggestive of hematometrocolpos, while pelvic MRI allowed precise localization of the obstruction and assessment of its extent [4,5]. These imaging findings were crucial in guiding management decisions and in avoiding aggressive manipulation in an infected and anatomically complex field.

Differential diagnosis

It is essential to distinguish this condition from other causes of painful primary amenorrhea, including isolated imperforate hymen, which is more common but without a deep vaginal anomaly. Mayer-Rokitansky-Küster-Hauser (MRKH) syndrome is characterized by congenital vaginal agenesis associated with the absence of a functional uterus and, consequently, does not result in hematometra. This entity should be distinguished from obstructive uterine malformations, such as isolated hematometra [6]. In addition, acquired conditions, including vaginal stenosis secondary to infection or trauma, must also be considered in the differential diagnosis of vaginal obstruction. This differentiation is crucial as it directs treatment and prognosis.

Therapeutic approaches and surgical challenges 

In our patient, therapeutic management was aimed at relieving the genital obstruction while minimizing the risk of complications and preserving future reproductive and sexual function. Preoperative counseling focused on explaining the objectives of treatment, namely, evacuation of retained menstrual blood and prevention of secondary complications, as well as the possibility of staged management depending on clinical evolution.

In this case, particular attention was given to the risk of postoperative complications. The patient was counseled regarding the possibility of infection despite antibiotic prophylaxis, bleeding, and especially restenosis, which represents the most frequent long-term complication and may require secondary corrective procedures such as Z-plasty [7]. Rare but severe complications, including vesicovaginal or rectovaginal fistulas, were also discussed. In addition, temporary therapeutic amenorrhea was prescribed as an adjunct to reduce menstrual flow during the postoperative period.

Management of our patient relied on surgical relief of the obstruction, with the dual objective of restoring a functional vaginal outflow to allow menstrual evacuation and preserving future fertility and sexual function. However, the presence of distal vaginal atresia combined with infection significantly increased the technical complexity of the procedure. Rather than proceeding directly to extensive reconstructive surgery, a conservative and staged approach was favored.

In this case, initial incision and drainage allowed safe evacuation of the hematocolpos and hematometra under antibiotic coverage, while limiting tissue trauma in an infected and anatomically distorted field. Definitive reconstructive surgery was deferred because tolerance to prolonged postoperative drainage and vaginal dilatation was considered limited, and the risk of restenosis was high in the acute setting.

Although several reconstructive techniques have been described in the literature, including puncture, resection, V-Y interdigitating flap plasty, and Z-plasty [8], these options were carefully weighed against the specific constraints of this patient. The final therapeutic strategy was therefore dictated by the patient's anatomy, clinical condition, and anticipated postoperative tolerance rather than by a predefined surgical algorithm.

Overall, this individualized approach was chosen to reduce the risk of complications while achieving effective decompression. Close long-term follow-up was planned to monitor healing, assess vaginal patency, and determine the need for secondary reconstructive intervention. Beyond anatomical outcomes, attention was given to the patient's quality of life and psychosexual well-being, recognizing the importance of comprehensive care in adolescent patients [9].

## Conclusions

The combination of a transverse vaginal septum and distal atresia is a rare but serious Müllerian anomaly. Early diagnosis using high-resolution imaging, such as MRI and ultrasound, is essential for precise anatomical assessment and surgical planning. Timely reconstructive surgery by experienced surgeons, with careful excision of the septum and management of the distal atretic segment, minimizes complications such as stenosis and adhesions.

With appropriate management, patients can achieve normal menstrual function, satisfactory sexual activity, and preserved fertility. High clinical suspicion in adolescents presenting with primary amenorrhea and cyclic pelvic pain is crucial to ensure early intervention and optimize functional and reproductive outcomes, highlighting the importance of specialized care in achieving the best prognosis.
